# Resveratrol Mitigates Oxygen and Glucose Deprivation-Induced Inflammation, NLRP3 Inflammasome, and Oxidative Stress in 3D Neuronal Culture

**DOI:** 10.3390/ijms231911678

**Published:** 2022-10-02

**Authors:** Ming-Chang Chiang, Christopher J. B. Nicol, Shy-Shyong Lo, Shiang-Wei Hung, Chieh-Ju Wang, Chien-Hung Lin

**Affiliations:** 1 Department of Life Science, College of Science and Engineering, Fu Jen Catholic University, New Taipei 242304, Taiwan; 2 Departments of Pathology and Molecular Medicine, Queen’s University, Kingston, ON K7L 3N6, Canada; 3 Departments of Biomedical and Molecular Sciences, Queen’s University, Kingston, ON K7L 3N6, Canada; 4 Cancer Biology and Genetics Division, Cancer Research Institute, Queen’s University, Kingston, ON K7L 3N6, Canada; 5 Division of Pediatric Immunology and Nephrology, Department of Pediatrics, Taipei Veterans General Hospital, Taipei 11217, Taiwan; 6 Department of Pediatrics, Zhongxing Branch, Taipei City Hospital, Taipei 10341, Taiwan; 7 Institute of Clinical Medicine, National Yang Ming Chiao Tung University, Taipei 11221, Taiwan; 8 College of Science and Engineering, Fu Jen Catholic University, New Taipei 242304, Taiwan

**Keywords:** resveratrol, oxygen-glucose deprivation, inflammation, oxidative stress, 3D scaffold

## Abstract

Oxygen glucose deprivation (OGD) can produce hypoxia-induced neurotoxicity and is a mature in vitro model of hypoxic cell damage. Activated AMP-activated protein kinase (AMPK) regulates a downstream pathway that substantially increases bioenergy production, which may be a key player in physiological energy and has also been shown to play a role in regulating neuroprotective processes. Resveratrol is an effective activator of AMPK, indicating that it may have therapeutic potential as a neuroprotective agent. However, the mechanism by which resveratrol achieves these beneficial effects in SH-SY5Y cells exposed to OGD-induced inflammation and oxidative stress in a 3D gelatin scaffold remains unclear. Therefore, in the present study, we investigated the effect of resveratrol in 3D gelatin scaffold cells to understand its neuroprotective effects on NF-κB signaling, NLRP3 inflammasome, and oxidative stress under OGD conditions. Here, we show that resveratrol improves the expression levels of cell viability, inflammatory cytokines (TNF-α, IL-1**β**, and IL-18), NF-κB signaling, and NLRP3 inflammasome, that OGD increases. In addition, resveratrol rescued oxidative stress, nuclear factor-erythroid 2 related factor 2 (Nrf2), and Nrf2 downstream antioxidant target genes (e.g., SOD, Gpx GSH, catalase, and HO-1). Treatment with resveratrol can significantly normalize OGD-induced changes in SH-SY5Y cell inflammation, oxidative stress, and oxidative defense gene expression; however, these resveratrol protective effects are affected by AMPK antagonists (Compounds C) blocking. These findings improve our understanding of the mechanism of the AMPK-dependent protective effect of resveratrol under 3D OGD-induced inflammation and oxidative stress-mediated cerebral ischemic stroke conditions.

## 1. Introduction

Today, stroke is the leading cause of long-term disability and death worldwide, resulting in a heavy burden on patients and society, especially in middle-income countries [1]. One in four adults will experience a stroke, and there are at least 10 million stroke survivors worldwide [2]. In addition, stroke can cause immediate neurological dysfunction and even lead to paralysis and severe patient death [3]. Therefore, many researchers focus on ischemic stroke because ischemic stroke is much higher than other types of stroke [4]. An ischemic stroke is primarily a temporary or permanent blockage or blood clot in an artery in the brain, further reducing blood flow to a specific brain area [5]. Insufficient blood supply causes brain cells to lose necessary glucose and oxygen, which disrupts the balance of the intracellular environment, triggers pathophysiological processes such as inflammation and oxidative stress, and ultimately leads to neuronal cell death and brain damage [6].

Cerebral ischemic stroke is usually caused by cerebrovascular occlusion, limited treatment options [7,8], lack of essential blood supply nutrients, and ultimately lead to neuronal cell death [7,8]. Several studies have focused on neuronal pathology, including inflammation, oxidative stress, and ischemic stroke leads to cell death [9,10,11]. Although the close link between hypoxia and inflammation and oxidative stress is well known, the basis of this link is not fully understood. Stroke-induced oxidative stress promotes the NF-κB signaling pathway [12]. The NLRP3 inflammasome has been reported to be the most well-studied inflammasome, and the role of the NLRP3 inflammasome in various diseases, such as cancer, diabetes, and neurodegenerative diseases [13,14]. However, information on the upregulation of NLRP3 will lead to a more robust inflammatory response in stroke is limited. These reactions lead to the initiation of the NLRP3 inflammasome, including NLRP3 (nucleotide-binding structural domain, leucine-rich family, containing pyridine structural domain 3), apoptosis-associated speckled protein (ASC) containing the caspase recruitment fraction, and caspase 1 [15,16]. This then promotes the activation of caspase 1, leading to neuronal apoptosis, which subsequently allows Pro-IL-1β and Pro-IL-18 to enter the activated IL-1β and IL-18, triggering an inflammatory response. Accumulating evidence suggests that the NLRP3 inflammasome promotes the secretion of excess proinflammatory cytokines, including IL-1β and IL-18, and leads to neuronal apoptosis [17]. In recent years, a variety of new information has emerged that oxidative stress is closely related to cerebrovascular disease, mainly from nerve inflammation, and accelerates the process of neuronal death [6,9]. Oxidative stress is not only caused by exogenous stimuli to generate reactive oxygen species (ROS), which can induce cell damage and apoptosis [18]. Nrf2 is a critical oxidative stress regulator protein that may have beneficial effects on ischemic stroke [19]. These potentially essential defense mechanisms may be associated with decreased expression of NF-kB and nlpk3 pathways and increased Nrf2 activity [20,21].

Resveratrol is a naturally occurring polyphenolic compound found in many plants, such as grapes and blueberries, capable of crossing the blood-brain barrier (BBB) [22,23]. Numerous studies have shown that resveratrol acts through AMPK signaling [24,25,26,27,28,29,30,31]. In vitro and in vivo experimental studies have shown that resveratrol can prevent or slow the progression of various diseases, including cerebrovascular disease, cancer, epilepsy, and pain [32,33]. However, the specific molecular mechanisms and roles in different diseases have not been fully elucidated. Resveratrol improves calorie restriction, insulin sensitivity, and aging and reduces inflammation, oxidative stress, mitochondrial dysfunction, and apoptosis [26,28,34,35,36,37]. Resveratrol can also be an anti-apoptotic agent, regulating various stages of programmed cell death. Resveratrol decreased the expression of some proteases, such as caspase-3 and caspase-9, and reduced the pro-apoptotic mediator Bax. This substance has neuroprotective effects against neurological diseases like stroke and neurodegenerative diseases [38,39,40]. Impaired AMPK activity occurs in mouse ischemic stroke and OGD cell models [24,41,42]. Several studies have observed that resveratrol may reduce neuronal atrophy and damage in the histological analysis of the brain in ischemic stroke [43,44]. Therefore, resveratrol has anti-inflammatory and antioxidant properties through AMPK signaling to achieve neuroprotective effects [34,45]. The molecular targeting and therapeutic efficacy of resveratrol depend on the activation of AMPK and SIRT1, inhibiting mTOR, NF-κB, and NLRP3 pathways [46,47]. Resveratrol has several additional targets, including Nrf2, COX, PDE, and PI3K. These findings suggest that resveratrol activates AMPK, reduces neuronal damage and apoptosis, and improves central nervous system function [48,49]. In addition, activation of AMPK by resveratrol affects neuronal energy homeostasis and contributes to neuroprotection [25]. However, there is controversy regarding resveratrol’s effects on disease and its side effects [50]. These studies lay the foundation for applying resveratrol in preventing ischemic stroke and OGD cell models [44,51,52].

Neurons in the brain, in particular, depend on the blood supply to maintain their oxygen and glucose needs. After an ischemic stroke, blood supply is reduced below a critical level, and brain tissue is affected by OGD. OGD of brain cells is the most common in vitro model of ischemic stroke and is widely used to study the cellular pathophysiology of ischemic stroke [53,54,55,56,57,58]. Numerous studies have reported OGD conditions mimicking an in vitro model of ischemic stroke in rat hippocampal slices [59], in primary cortical neurons [60], SH-SY5Y cells [61], HT-22 cells [54], PC-12 cells [62], BV2 microglia [63], and human astrocytes [64]. 3D structures can better simulate cell behavior and key organizational features in vivo, which is a challenge, especially for neural tissue applications [65]. 3D cell culture is a platform for cell growth and propagation, and appropriate 3D scaffolds can be combined with cells to develop biomimetic tissues [66]. Gelatin is one of the natural polymers with excellent biocompatibility and tissue-like texture, and the construction of gelatin-rich 3D constructs supports the integration of cell adhesion and proliferation [67]. Many studies demonstrate that gelatin scaffolds build a 3D platform to mimic the extracellular matrix to provide mechanical support for cells [67,68]. 3D cell culture technology provides a more realistic in vivo microenvironment and has been used as a model for disease and ischemic pathological conditions [69,70,71]. Our previous studies have shown that 3D gelatin scaffolds can be used in SH-SY5Y cells [61] and human neural stem cells [72], constructing OGD and Alzheimer’s disease models for studying mitochondrial dysfunction. Therefore, in this study, we investigated the role of resveratrol in 3D gelatin scaffold cells to understand its neuroprotective effects against NF-κB signaling, NLRP3 Inflammasome, and oxidative stress under OGD conditions.

## 2. Results

### 2.1. Resveratrol Rescues OGD-Mediated Cell Viability and TNF-α under the 3D Scaffold

**Figure 1:**
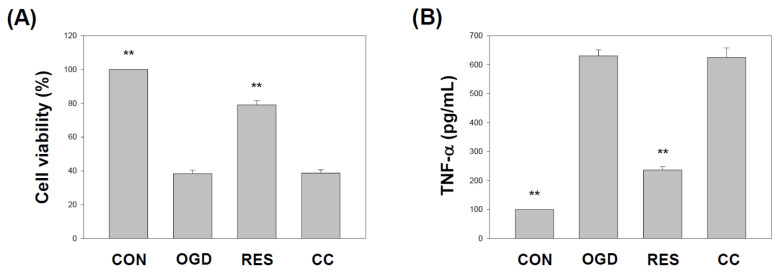
Resveratrol rescues OGD-mediated cell viability and TNF-α under the 3D scaffold. The cells were treated with OGD under the scaffold for 24 h and then treated with 10 μM resveratrol (AMPK activator) or 10 μM Compound C (AMPK antagonist) for another 48 h. The cell experiment under the scaffold was divided into four groups: (1) Control (CON) group represents cells with no treatment, cultured in a new medium for 72 h; (2) OGD group represents cells treated with OGD for 24 h, then exchanged with the new medium for another 48 h; (3) Resveratrol (RES) group, represents cells treated with OGD for 24 h, then exchanged the new medium with 10 μM Resveratrol for another 48 h; and (4) Compound C (CC) group represents cells treated with OGD for 24 h, then treated then exchanged the new medium with 10 μM Compound C and 10 μM Resveratrol for another 48 h. (**A**) Cell viability was detected by an SRB assay. (**B**) The cell culture supernatant was harvested, and ELISA measured the secretion of TNF-α. Values are expressed as percentages of the indicated level in CON and are presented as the mean ± SEM values from three independent experiments. Specific comparison to the indicated SH-SY5Y cells with OGD ** *p* < 0.001 vs. cells with OGD.

First, the effects of OGD on SH-SY5Y cell viability under the 3D scaffold (Figure S2) were assessed by the SRB assay. Compared to vehicle controls, the cells treated with OGD show significantly decreased cell survival (Figure 1A). In addition, the effect of 10 μM resveratrol and 10 μM compound C in SH-SY5Y cells is based on our previously published research [61]. Furthermore, treatment with resveratrol significantly rescued cell survival, although this protective effect was blocked by co-treatment with an antagonist of AMPK (Compound C) (Figure 1A). Inflammation may severely alter the viability of neuronal cells [73,74,75]. Therefore, the treated SH-SY5Y cells were evaluated for changes in the secretion levels of pro-inflammatory cytokines by ELISA. Treatment with resveratrol significantly normalized the increase in OGD-induced tumor necrosis factor-α (TNF-α) secretion in SH-SY5Y cells (Figure 1B). Compound C co-treatment blocked this effect.

### 2.2. Resveratrol Normalizes the Expression of IKK and NF-κB (p65) in OGD-Treated Cells on the 3D Scaffold

**Figure 2:**
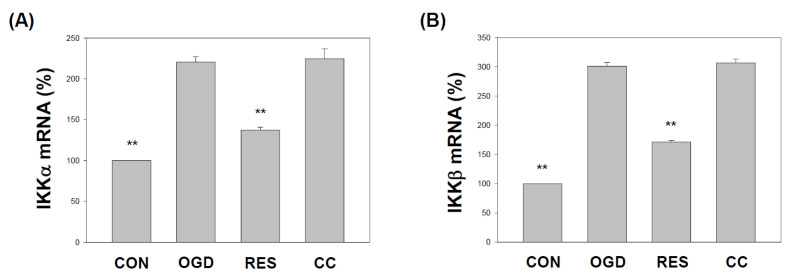
Resveratrol rescues IKKα and IKKβ mRNA expression in OGD-induced cells within a 3D scaffold. The cell experiments under the scaffolds were divided into four groups, as described in Figure 1 and the methods section. Use qPCR to analyze IKKα (**A**) and IKKβ (**B**) mRNA transcripts. Collect total RNA from SH-SY5Y cells and reverse transcribe it into cDNA. Next, perform qPCR on the specified gene and normalize it to GAPDH expression. The value is expressed as a percentage of the transcript established in CON and described as the mean ± SEM value from three independent experiments. Specific comparison to the indicated SH-SY5Y cells with OGD ** *p* < 0.001 vs. cells with OGD.

**Figure 3:**
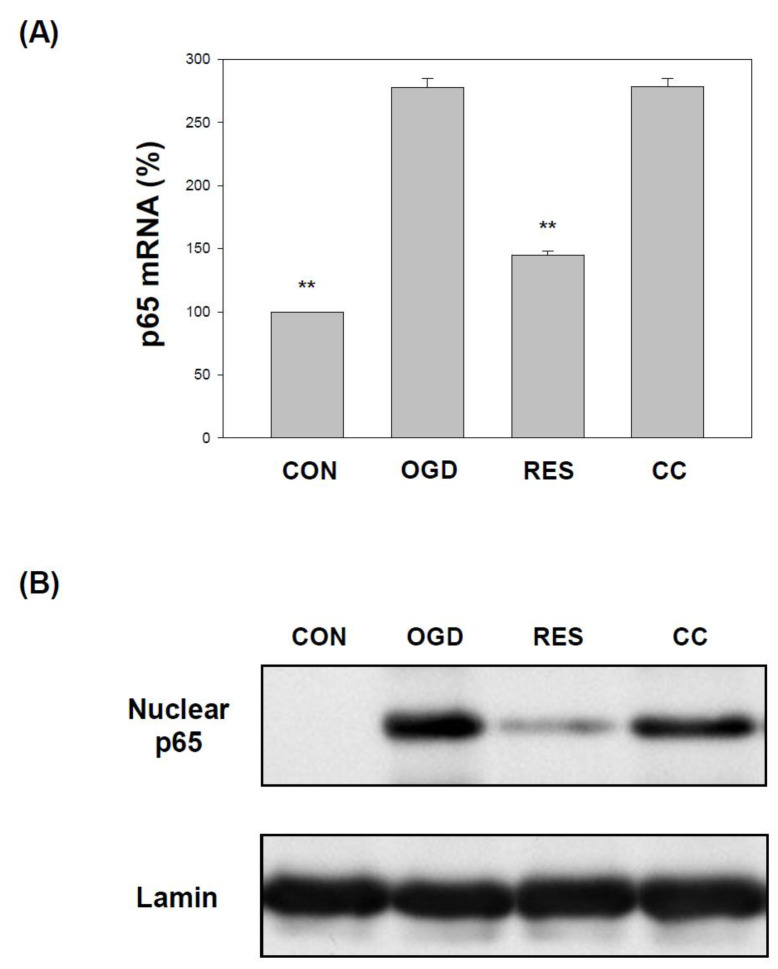
Resveratrol normalizes p65 levels in the OGD-induced cells in the 3D scaffold. The cell experiments under the scaffolds were divided into four groups, as described in Figure 1 and the methods section. (**A**) qPCR was used to analyze the p65 mRNA level in each treatment group. Collect total RNA from cells and reverse transcribe it into cDNA. Perform qPCR on the specified gene and normalize it to GAPDH expression. The value is expressed as a percentage of the transcript established in CON and the mean ± SEM value from three independent experiments. (**B**) Collect nuclear components (20 μg per lane) from the specified conditions and perform the level of p65 protein by a Western blot analysis. The value is expressed as the percentage of SH-SY5Y cells treated with OGD and described as the mean ± SEM value from three independent experiments. Specific comparison to the indicated SH-SY5Y cells with OGD ** *p* < 0.001 vs. cells with OGD.

OGD induces neuroinflammation in hippocampal neurons [76], PC12 cells [77], human BE(2)M17 neuroblastoma cells [78], and increases NF-κB pathway signaling [79]. This results in increased transcription of downstream target genes [80], leading to increased levels of inflammatory chemokines and cytokines [81,82]. Therefore, we evaluated the levels of the IKKα, IKKβ, and p65 transcripts in cells exposed to OGD. We found that the mRNA transcripts of IKKα, IKKβ, and p65 were higher in the SH-SY5Y cells treated with OGD (Figure 2 and Figure 3A). Treatment with resveratrol significantly normalized the expression of IKKα, IKKβ, and p65, but only in the absence of co-treatment with compound C (Figure 2 and Figure 3A). In addition, the incubation of cells with OGD induced the translocation of p65 protein into the nucleus (Figure 3B and Figure S3A). Compared with the OGD treatment alone, co-treatment with resveratrol significantly normalized p65 nuclear expression in the cells, but these changes were not present in the presence of Compound C (Figure 3B and Figure S3A).

### 2.3. Resveratrol Normalizes NLRP3 Inflammasome Levels in OGD-Induced Cells in the 3D Scaffold

**Figure 4:**
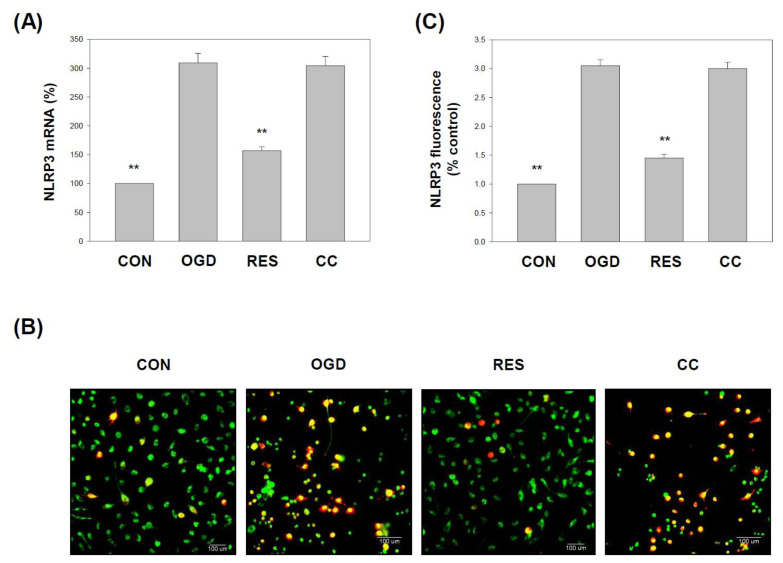
Resveratrol normalizes NLRP3 levels in OGD-induced cells in the 3D scaffold. The cell experiments under the scaffolds were divided into four groups, as described in Figure 1 and the methods section. (**A**) qPCR was used to analyze the NLRP3 mRNA level in each treatment group. Collect total RNA from the cells and reverse transcribe it into cDNA. Perform qPCR on the specified gene and normalize it to GAPDH expression. (**B**) Immunostaining of the cells was performed using anti-NLRP3 and anti-MAP2 antibodies. NLRP3 was visualized using the Avidin-Alexa Fluor^®^ 568 (red)-conjugated secondary antibody. MAP2 was visualized using Avidin-Alexa Fluor^®^ 488 (green)-conjugated secondary antibody. A representative image of three independent experiments is shown. Scale bar: 100 μm. (**C**) The fluorescence intensity of the immunostaining was quantified. The value is expressed as a percentage of the transcript established in CON and the mean ± SEM value from three independent experiments. Specific comparison to the indicated SH-SY5Y cells with OGD ** *p* < 0.001 vs. cells with OGD.

Growing evidence points to the importance of NLRP3 inflammasome activation in ischemic stroke inflammation [17]. NLRP3 has emerged as a critical mediator of ischemic inflammation, leading to the final response of the inflammatory cascade and neuronal cell death [83]. Here, the expression of NLRP3 was significantly increased in the OGD-treated cells compared to the respective vehicle controls (Figure 4). Cotreatment with resveratrol increased NLRP3 mRNA (Figure 4A) levels in cells compared to the OGD-treatment alone. Immunofluorescence staining of treated cells for NLRP3 and neuronal marker MAP2 (microtubule-associated protein 2) are shown in Figure 4B.

The levels of NLRP3 were higher in OGD-exposed cells than in the respective vehicle controls (Figure 4C). Cotreatment with resveratrol significantly decreased NLRP3 expression levels in cells compared to the OGD treatment alone (Figure 4)

**Figure 5:**
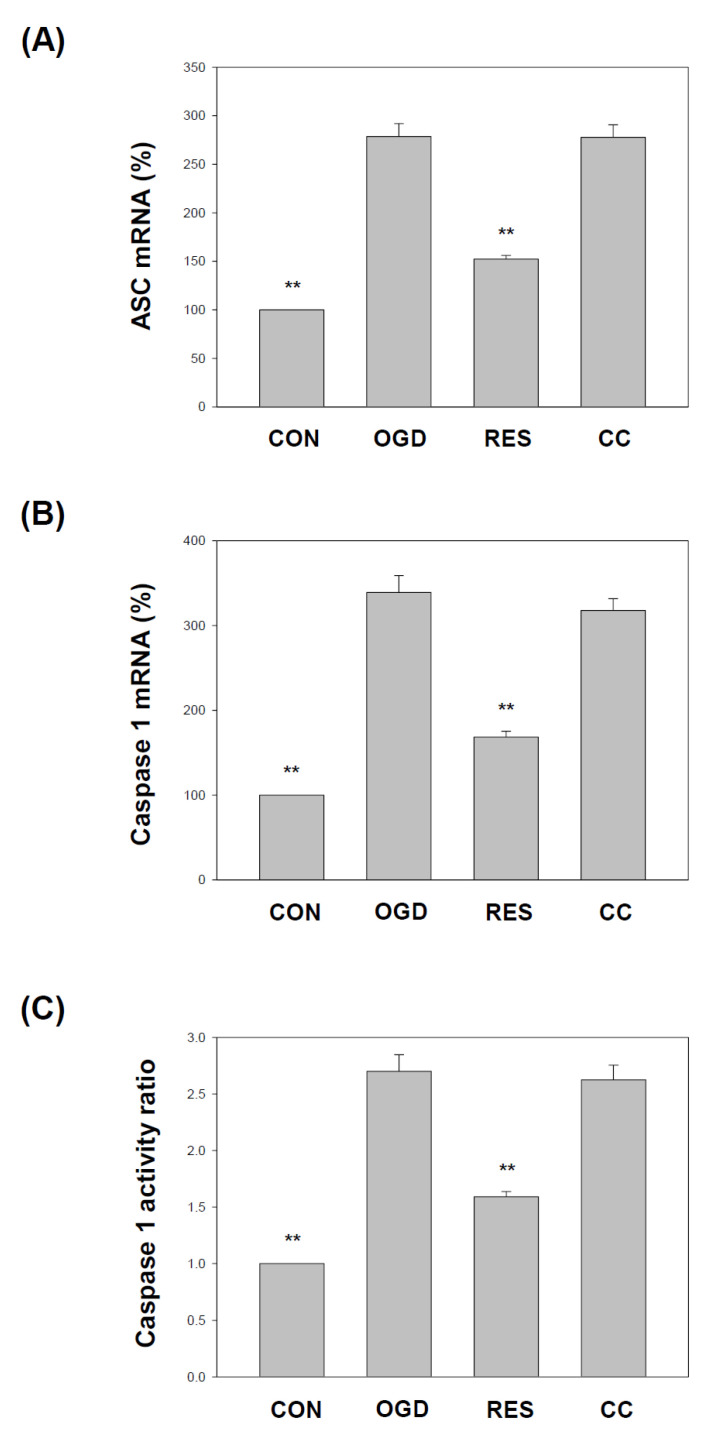
Resveratrol improves the ASC and Caspase 1 levels in OGD-induced cells in the 3D scaffold. The cell experiments under the scaffolds were divided into four groups, as described in Figure 1 and the methods section. Use qPCR to analyze ASC (**A**) and Caspase 1 (**B**) mRNA transcripts. Collect total RNA from SH-SY5Y cells and reverse transcribe it into cDNA. Perform qPCR on the specified gene and normalize it to GAPDH expression. (**C**) Caspase 1 activity was detected by a fluorometric protease assay using substrate YVAD-AFC (AFC: 7-amino-4-trifluoromethyl coumarin). The value is expressed as a percentage of the transcript established in CON and the mean ± SEM value from three independent experiments. Specific comparison to the indicated SH-SY5Y cells with OGD ** *p* < 0.001 vs. cells with OGD.

**Figure 6:**
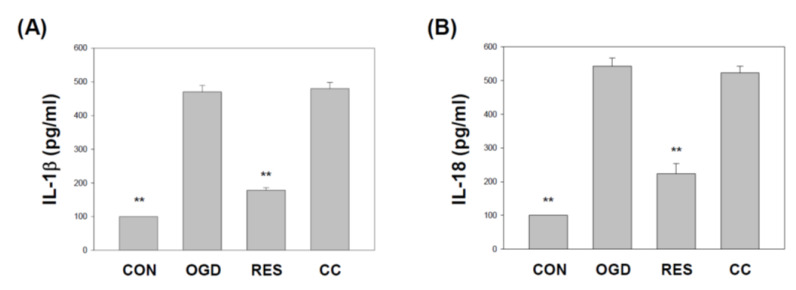
Resveratrol normalizes OGD-induced of IL-1β and IL-18 under the 3D scaffold. The cell experiments under the scaffolds were divided into four groups, as described in Figure 1 and the methods section. The cell culture supernatant was harvested, and ELISA measured the secretion of IL-1β (**A**) and IL-18 (**B**). Values are expressed as percentages of the indicated level in CON and are presented as the mean ± SEM values from three independent experiments. Specific comparison to the indicated SH-SY5Y cells with OGD ** *p* < 0.001 vs. cells with OGD.

The NLRP3 inflammasome contains ASC and caspase 1. Both are essential adaptor proteins, and pro-apoptotic molecules of the inflammasome serve as a platform for activating caspase 1 [84]. We showed that compared with the corresponding vehicle control, the levels of apoptosis-associated speck-like protein containing a caspase recruitment domain (ASC) and caspase 1 (Figure 5) in SH-SY5Y cells treated with OGD were significantly reduced. Compared with OGD treatment alone, co-treatment with resveratrol increased ASC mRNA (Figure 5A), caspase 1 mRNA (Figure 5B), and caspase 1 activity (Figure 5C) levels in cells. Still, the effect of this resveratrol was affected by the Compound C block. The NLRP3 inflammasome regulates the release of pro-inflammatory factors IL-1β and IL-18, one of the critical steps leading to neuronal cell death associated with ischemic stroke [17]. In the present study, the treated cells were evaluated using an enzyme-linked immunosorbent assay for changes in the levels of proinflammatory cytokine secretion. Cotreatment with resveratrol significantly decreased IL-1β and IL-18 expression levels in cells compared to OGD treatment alone (Figure 6).

### 2.4. Resveratrol Rescues Oxidative Stress in OGD-Treated Cells on a 3D Scaffold

**Figure 7:**
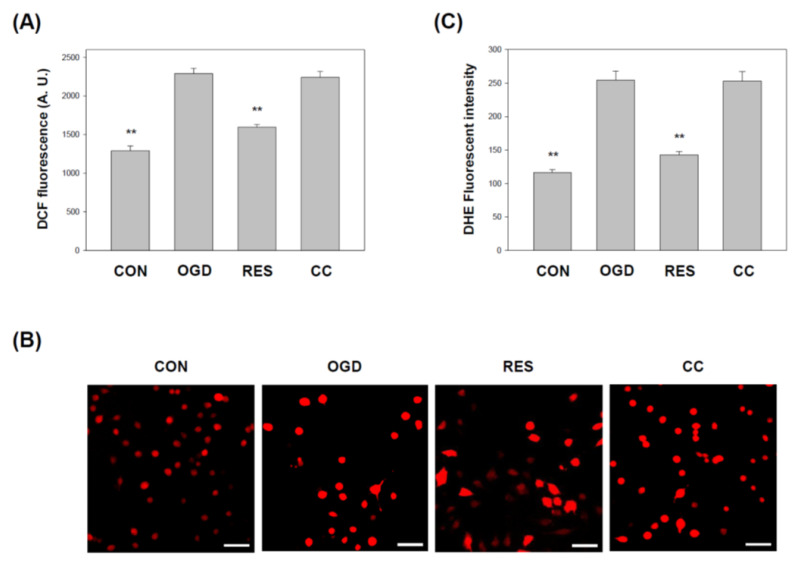
Resveratrol normalized cell-induced oxidative stress by OGD in 3D scaffolds. The cell experiments under the scaffolds were divided into four groups, as described in Figure 1 and the methods section. (**A**) The lysate harvested from the specified conditions was subjected to ROS determination by DCFH-DA. (**B**) Collect cells to show a typical photomicrograph of DHE dye (red). Scale bar: 100 μm. (**C**) Quantitative data generated by ROS evaluated by DHE fluorescence intensity is normalized to cell number data. Values are expressed as percentages of the indicated level in CON and are presented as the mean ± SEM values from three independent experiments. Specific comparison to the indicated SH-SY5Y cells with OGD ** *p* < 0.001 vs. cells with OGD.

OGD is induced oxidative stress in HT-22 cells [54] and PC-12 cells [62]. The oxidative stress in the cells was further assessed by DCF and DHE staining assays. DCF fluorescence analysis showed that the ROS level in OGD-treated cells (Figure 7A) was significantly increased compared to the control. Furthermore, compared with the control, the DHE staining in SH-SY5Y cells treated with OGD also increased significantly (Figure 7B,C). In contrast, resveratrol treatment normalizes oxidative stress, but this effect is prevented by Compound C.

### 2.5. Resveratrol Increases Nrf2 Expression in OGD-Treated Cells on 3D Scaffolds

**Figure 8:**
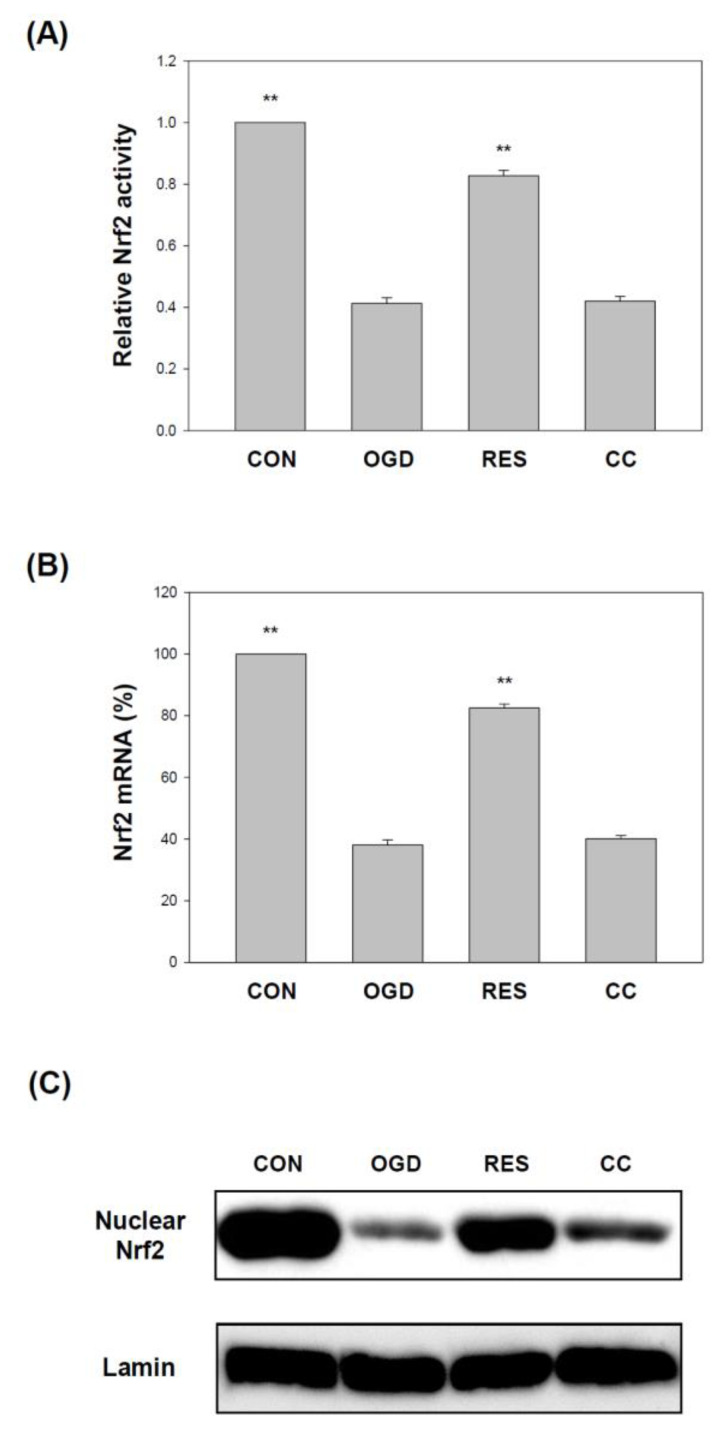
Resveratrol normalized Nrf2 activity and genes by OGD in 3D scaffolds. The cell experiments under the scaffolds were divided into four groups, as described in Figure 1 and in the methods section. The Nrf2 level was detected by the Nrf2 transcription factor assay (**A**). (**B**) qPCR was used to analyze the Nrf2 mRNA level in each treatment group. (**C**) Collect nuclear components (20 μg per lane) from the specified conditions and perform the level of Nrf2 protein by Western blot analysis. Values are expressed as percentages of the indicated level in CON and are presented as the mean ± SEM values from three independent experiments. Specific comparison to the indicated SH-SY5Y cells with OGD ** *p* < 0.001 vs. cells with OGD.

Nrf2 is an important part of cellular defense and survival pathways against oxidative stress [57,85]. Nrf2 has recently attracted more and more attention in ischemic stroke [86]. It has been reported that the expression of Nrf2 is reduced in stroke models [87,88], and Nrf2 inhibition is associated with increased inflammation and oxidative stress [89,90,91]. Here, compared with the corresponding vector control, the expression of Nrf2 in OGD-treated cells was significantly reduced (Figure 8). Compared with OGD treatment alone, co-treatment with resveratrol increased Nrf2 activity (Figure 8A), mRNA (Figure 8B), and protein (Figure 8C and Figure S3B) levels in cells, but the effect of this resveratrol was affected by the Compound C block.

### 2.6. Resveratrol Increases the Expression of SOD, Gpx, GSH, Catalase, and HO-1 in OGD-Treated Cells on the 3D Scaffold

**Figure 9:**
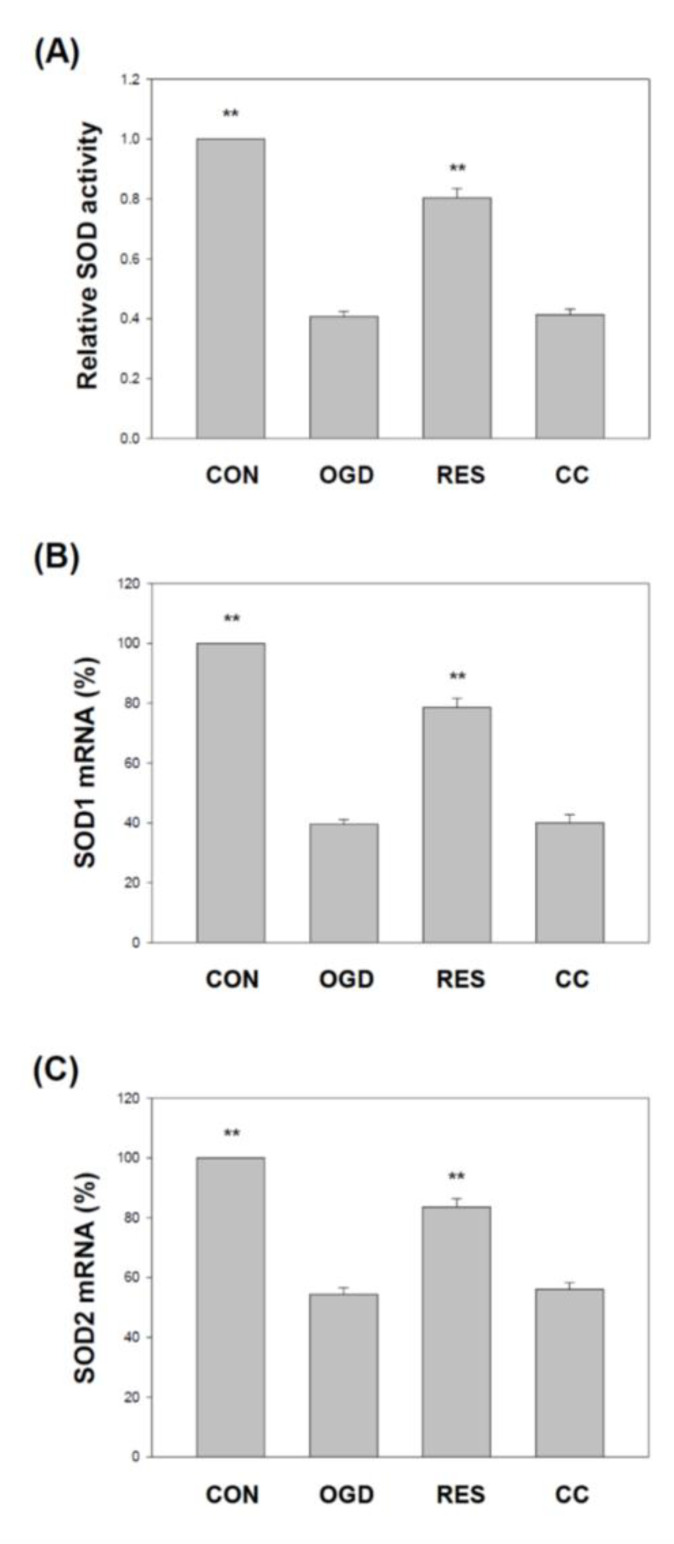
Resveratrol rescues SOD activity and gene expression by OGD in 3D scaffolds. The cell experiments under the scaffolds were divided into four groups, as described in Figure 1 and the methods section. SOD activity was detected by a SOD activity assay (**A**). qPCR was used to analyze each treatment group’s SOD1 (**B**) and SOD2 (**C**) mRNA levels. Values are expressed as percentages of the indicated level in CON and are presented as the mean ± SEM values from three independent experiments. Specific comparison to the indicated SH-SY5Y cells with OGD ** *p* < 0.001 vs. cells with OGD.

**Figure 10:**
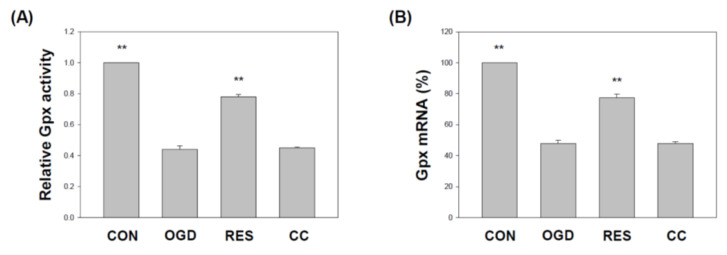
Resveratrol rescues Gpx activity and gene expression by OGD in 3D scaffolds. The cell experiments under the scaffolds were divided into four groups, as described in Figure 1 and the methods section. Gpx activity was detected by the Gpx activity colorimetric method (**A**). (**B**) qPCR was used to analyze the Gpx mRNA level in each treatment group. Values are expressed as percentages of the indicated level in CON and are presented as the mean ± SEM values from three independent experiments. Specific comparison to the indicated SH-SY5Y cells with OGD ** *p* < 0.001 vs. cells with OGD.

**Figure 11:**
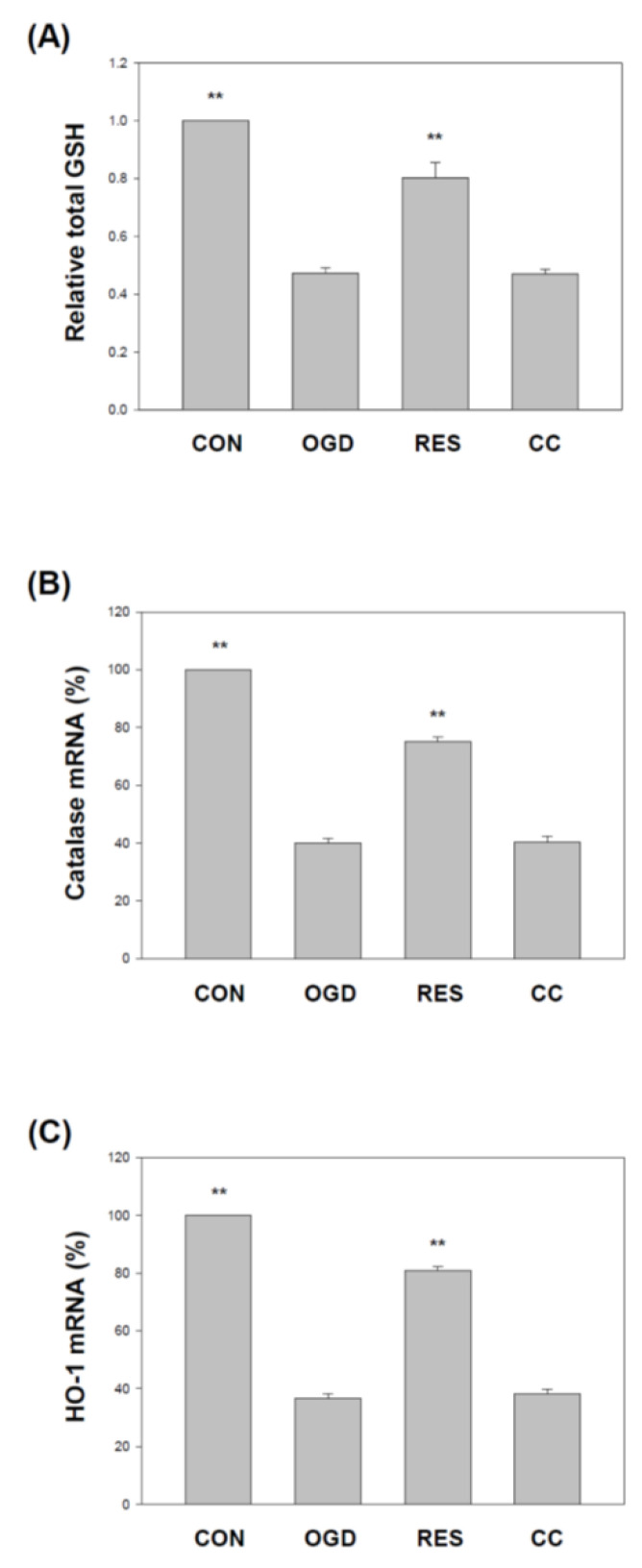
Resveratrol normalized GSH levels, catalase, and HO-1 gene expression by OGD in 3D scaffolds. The cell experiments under the scaffolds were divided into four groups, as described in Figure 1 and the methods section. GSH levels were detected using the Glutathione Colorimetric Assay Kit (**A**). qPCR was used to analyze each treatment group’s catalase (**B**) and HO-1 (**C**) mRNA levels. Values are expressed as percentages of the indicated level in CON and are presented as the mean ± SEM values from three independent experiments. Specific comparison to the indicated SH-SY5Y cells with OGD ** *p* < 0.001 vs. cells with OGD.

Several studies have reported reductions in superoxide dismutase (SOD) and glutathione peroxidase (Gpx) in various stroke models, including H9c2 [92,93], PC12 cells [93], and hippocampal neuronal cells [94]. We showed that compared with the corresponding vehicle control, the levels of SOD (Figure 9) and GPx (Figure 10) in the SH-SY5Y cells treated with OGD were significantly reduced. Compared with the OGD treatment alone, co-treatment with resveratrol increased the SOD1, SOD2, and GPx in the cells, but this resveratrol effect was blocked by Compound C (Figure 9 and Figure 10). In addition, glutathione (GSH) [95], catalase [92] and heme oxygenase-1 (HO-1) [96] reduced levels in various stroke models. We showed that compared with the corresponding vehicle control, the GSH, catalase, and HO-1 in OGD-treated cells were reduced (Figure 11). Compared with OGD treatment alone, co-treatment with resveratrol increased the GSH, catalase, and HO-1 in the cells, but this resveratrol effect was blocked by Compound C (Figure 11).

## 3. Discussion

The in vivo middle cerebral artery occlusion (MCAO) animal model and the in vitro OGD stroke model are used to study the mechanism of ischemic stroke induction by activating the NF-κB pathway, NLRP3 inflammasome, and triggering a vicious cycle of oxidative stress, leading to neurotoxicity [95,97]. Neuroinflammation and oxidative stress are the main reasons for the morbidity and mortality of brain injury caused by stroke. Resveratrol has been shown to activate the AMPK pathway, which may be a therapeutic target for stroke [24]. In addition, 3D models form biomimetic tissues that mimic the body’s microenvironmental conditions [98]. Cells cultured in 3D biomaterials can become extracellular matrices to support cell growth or cell interactions to form new biomimetic tissues [99]. Gelatin is one of the most commonly used natural biopolymer scaffolds for a 3D cell culture, offering suitable biocompatibility [67]. Here, we summarized the effect of resveratrol on the 3D gelatin scaffold in cells during GD-induced inflammation (Figure 1), IKK and NF-κB cascade in cells (Figure 2 and Figure 3), NLRP3 inflammasome (Figure 4, Figure 5 and Figure 6), oxidative stress (Figure 7), and antioxidant gene expression (Nrf2, SOD, Gpx, GSH, catalase, and HO-1) (Figure 8, Figure 9, Figure 10 and Figure 11).

Inflammation is implicated in many disorders, particularly inflammatory diseases and ischemic stroke, where ischemia leads to tissue hypoxia and induces an inflammatory phenotype [9]. At the molecular level, NF-κB plays a crucial role in the mechanism between hypoxia and inflammation [100]. However, the inflammatory response to hypoxia depends on NF-κB and NLRP3 inflammasome, so the molecular mechanisms behind these connections remain further investigated. Brain inflammatory responses and NLRP3 inflammasome are activated after ischemic stroke, triggering neuronal cell damage, brain edema, and neurological dysfunction [83,101]. NLRP3 inflammasome is activated under OGD conditions, increasing the release of caspase 1-dependent proinflammatory cytokines, such as IL-1β and IL18 [102]. Because persistent inflammation exacerbates post-OGD stroke brain damage, anti-inflammatory drugs that reduce NLRP3 inflammasome activation may be neuroprotective [103]. In animal models, inhibition of NLRP3 inflammasome reduces neuron death and infarct volume [102,103].

In 2012, Shin et al. reported the neuroprotective effect of resveratrol on the ischemic cortex [104]. In addition, a 2018 study showed the anti-inflammatory effects of resveratrol on cerebral ischemic injury in MCAO rats [105]. Recently, Liu et al. demonstrated that resveratrol might inhibit cerebral ischemia by inducing inflammatory cytokines and neuroinflammation in MCAO mice [106]. Le et al. showed that resveratrol exhibits an anti-neuro-inflammatory mechanism in mouse BV2 microglia after exposure to OGD [107]. Another study by Liu et al. indicated that resveratrol attenuates neuroinflammation and inhibits the NF-κB pathway in the OGD model of microglia [108]. Several works of the literature suggest that the anti-neuroinflammatory effects of resveratrol may have protective effects on the brain, related to inhibition of NLRP3 inflammasome activation [109,110]. Some studies indicate that resveratrol can inhibit the activation of the NLRP3 inflammasome and the subsequent inflammatory response in ischemic stroke [17,111]. Our findings indicate that resveratrol protects cells from OGD injury by inhibiting the inflammatory response mediated by the NF-κB pathway (Figure 1, Figure 2 and Figure 3) and NLRP3 inflammasome activation (Figure 4, Figure 5 and Figure 6).

Oxidative stress is the key pathogenic mechanism of ischemic stroke [112,113]. Some findings suggest that resveratrol can reduce OGD-induced ROS, oxidative stress, and apoptosis and improve cell survival [114,115,116]. Furthermore, resveratrol can boost antioxidant defenses such as Nrf2, SOD, Gpx GSH, catalase, and HO-1, confirming it is an antioxidant [47,117]. Li et al. showed that resveratrol reduces infarct volume and cerebral edema by improving oxidative stress and increasing SOD expression in rats with cerebral ischemic injury [118]. Liu and his collaborators showed that resveratrol might affect PC12 cells by regulating SOD and catalase activities and OGD-induced apoptosis [115]. In addition, resveratrol may activate Nrf2 related to oxidative stress through AMPK, thereby inducing the expression of more than 500 genes involved in the antioxidant system [87,119,120,121]. Recent studies have shown that the activation of AMPK and Nrf2 pathways modulate antioxidant effects against ischemic stroke through the SOD and HO-1 [85,122]. Ren et al. showed that resveratrol reduces ischemic injury by increasing the expression of Nrf2 in rats (Ren J et al., 2011). In 2018, three studies showed that resveratrol reduces the oxidative stress caused by hypoxia in rats through the Nrf2 pathway [123] and reduces the oxidation of ischemic injury in rats through the Nrf2/NF-κB pathway stress [124] by enhancing the Nrf-2 signal in primary cortical neurons [123]. Our findings suggest that the neuroprotective effect of resveratrol in OGD is achieved through multiple pathways, including direct scavenging of reactive oxygen species (Figure 7) as antioxidants and activation of the Nrf2 response pathway (Figure 8), and rescue of endogenous antioxidant genes (Figure 9, Figure 10 and Figure 11).

Inflammatory responses and ROS in the brain play essential roles in the progression of secondary injury after ischemic stroke [12]. Therefore, appropriate inflammatory and oxidative stress regulation may have an important role. The NF-κB and NLRP3 inflammasomes are required to initiate inflammation, and the effects of ROS may also be Nrf2-dependent, and both are implicated in the mechanism of ischemic stroke [20,83]. This study aimed to investigate the neuroprotective effect of resveratrol via AMPK in an OGD model and to elucidate the underlying mechanism by which cells display resveratrol-related regulation on 3D scaffolds. Our findings demonstrate that resveratrol inhibits the activation of the NF-κB and NLRP3 inflammasomes and reduces the production of inflammatory cytokines. The effect of resveratrol on reducing ROS and oxidative stress may be through Nrf2 and its downstream antioxidant genes. Furthermore, these protective effects of resveratrol were blocked by the AMPK inhibitor Compound C. Therefore, our findings may extend the critical role of resveratrol in neuroprotection via AMPK and may help treat ischemic stroke patients.

## 4. Materials and Methods

### 4.1. Cell Culture

Human SH-SY5Y neuroblastoma cells are a human-derived cell line widely used in neuroscience as an in vitro model for studying neuronal function and differentiation [125]. We also have studies on the OGD model [61] and PM2.5 toxicity [126] using SH-SY5Y cells. SH-SY5Y cells, originally obtained from the American Type Culture Collection (Manassas, VA, USA), were maintained and supplemented with 10% FBS (Invitrogen) plus 2 mM L-glutamine and 1% penicillin/streptomycin (Invitrogen) in an incubation room inflated with 5% CO2/95% air at 37 °C. Cellular morphology and expression of the neuronal marker MAP2 in the SH-SY5Y cells are presented in Figure S1. SH-SY5Y cells were exposed to oxygen-glucose deprivation (OGD) in 3D gelatin scaffolds (Figure S2; height: 2 mm, diameter: 8 mm; HyCell International Co), as reported elsewhere in our study [62]. Briefly, to induce OGD in vitro, SH-SY5Y cells were washed twice with pre-warmed PBS and then in an O_2_/N_2_/CO_2_ incubator (ASTEC CO LTD, Fukuoka, Japan)

glucose-free and serum-free DMEM culture and exposed to 1% O_2_ for a specified period. For 3D states, the cells were exposed to a 1% O_2_ incubator for 24 h under the 3D gelatin scaffold, and then we used the specified reagents (Resveratrol and Compound C, both from Sigma) for another 48 h. The cells were treated with OGD under the scaffold for 24 h and then treated with 10 μM resveratrol (AMPK activator) or 10 μM Compound C (AMPK antagonist) for another 48 h. The cell experiment under the scaffold was divided into four groups: (1) Control (CON) group represents cells with no treatment, cultured in a new medium for 72 h; (2) OGD group represents cells treated with OGD for 24 h, then exchanged with the new medium for another 48 h; (3) Resveratrol (RES) group, represents cells treated with OGD for 24 h, then exchanged with the new medium with 10 μM resveratrol for another 48 h; and (4) Compound C (CC) group represents cells treated with OGD for 24 h, then treated and exchanged the new medium with 10 μM Compound C and 10 μM resveratrol for another 48 h. The effect of resveratrol on the SH-SY5Y cells within a 3D scaffold was analyzed using an MTT assay. The effect of resveratrol exposure for 48 h on the cell viability of SH-SY5Y cells is shown in Figure S4, which indicates that the use of 20 μM or higher concentrations of resveratrol results in significant cell death. Therefore, a lower concentration of 10 μM resveratrol was chosen for all cell experiments. The use of resveratrol in cells at a concentration of 10 μM and Compound C in cells at a concentration of 10 μM are based on our previous paper [61], which has also been validated.

### 4.2. Assessment of Cell Viability and Interleukin

Cells were exposed to OGD on a 3D gelatin scaffold for 24 h, then treated with the indicated reagents for another 48 h. Cell viability was assessed using an in vitro Sulforhodamine B (SRB)-based assay kit (Sigma, Austin, TX, USA) using an ELISA Reader. In addition, the concentrations of interleukins were assessed using supernatants obtained from cell culture supernatant as indicated. The ELISA assay (R&D Systems, Abingdon, UK) measured the secretion of TNF-α, IL-1β, and IL-18, and the absorbance was read at 450 nm according to the manufacturer’s instructions.

### 4.3. Quantitative Polymerase Chain Reaction

Total RNA will be isolated using the TriReagent kit and then transcribed into cDNA using Superscript* II reverse transcriptase (Thermo Fisher Scientific Inc., Waltham, MA, USA). Next, we used the TaqMan kit (Applied Biosystems, Waltham, MA, USA) to perform quantitative PCR (qPCR) on the StepOne quantitative PCR machine as described elsewhere [127]. The primer sequence is as follows:

IKKα (F: 5′-GAAGGTGCAGTAACCCCTCA-3′ and R: 5′-ATTGCCCTGTTCCTCATT-TG-3′), IKKβ (F: 5′-AGCATGAATGCCTCTCGACT-3′ and R: 5′-TTCTAGCAGGGT-GCAGAGGT-3′), p65 (F: 5′-ATGGCTTCTATGAGGCTGAG-3′ and R: 5′-GTTGTTGT-TGGTCTGGATGC-3′), NLRP3 (F: 5′-TGCCCGTCTGGGTGAGA-3′ and R: 5′-CCGG-TGCTCCTTGATGAGA-3′), ASC (F: 5′-CGCGAGGGTCACAAACGT-3′ and R: 5′-T-GCTCATCCGTCAGGACCTT-3′), caspase-1 (F: 5′-AATTTTCCGCAAGGTTCGATT-3′ and R: 5′-ACTCTTTCAGTGGTGGGCATCT-3′), Nrf2 (F: 5′-TCAGCCAGCCCAGC-ACATCC-3′ and R: 5′-TCTGCGCCAAAAGCTGCATGC-3′), SOD1 (F: 5′-AAGGCC-GTGTGCGTGCTGAA-3′ and R: 5′-CAGGTCTCCAACATGCCTCT-3′); SOD2 (F: 5′--GCACATTAACGCGCAGTCA-3′ and R: 5′-AGCCTCCAGCAACTCTCCTT-3′); Gpx (F: 5′-CCTCAAGTACGTCCGACCTG-3′ and R: 5′-CAATGTCGTTGCGGCACACC-3′); Catalase (F: 5′-TGGGATCTCGTTGGAAATAACAC-3′ and R:5′-TCAGGACGTAGG-CTCCAGAAG-3′); HO-1 (F: 5′-AAGACTGCGTTCCTGCTCAAC-3′ and R: 5′-AAAG-CCCTACAGCAACTGTCG-3′) and GAPDH (F: 5′-TGCACCACCAACTGCTTAGC-3′ and R: 5′-GGCATGGACTGTGGTCATGAG-3′).

### 4.4. Western Blot (WB) Detection

The cell lysate (20 μg) was collected from the specified conditions and analyzed by WB. Equal amounts of protein were separated by SDS–PAGE using 10% polyacrylamide gels according to the protocol’s methods [72]. The cells transferred to the PVDF membrane were blocked with cell signaling technology primary antibodies against p65 (1:1000 dilution), Nrf2 (1:2000 dilution), MAP2 (1:3000 dilution), and lamin (1:3000). After reacting with the secondary antibody, we used an enhanced chemiluminescence reagent (Millipore) to observe specific protein bands, and used the ImageJ software (Version 1.53t) to quantify the results.

### 4.5. ROS Measurement by Staining with Dichlorofluorescein Diacetate (DCFH-DA) and Dihydroethidium (DHE)

First, the level of intracellular ROS was detected by DCFH-DA: the cells were placed in a 6-well culture plate at a density of 2 × 10^5^ per well. Next, the cells were washed with PBS, followed by PBS containing 10 μg/μL DCFH-DA (Molecular Probes Inc., Eugene, OR, USA). Plates were read for 1 hr in a microplate reader with excitation at 485 nm and emission at 530 nm, as previously described [128]. In addition, intracellular ROS was measured by DHE staining: the cells were loaded into 6-well culture plates at a density of 2 x10^5^ per well. Finally, cells were incubated with complete StemPro NSC SFM without phenol red containing 5 μM DHE (Molecular Probes Inc.). After 1 hr, the cells were imaged under a fluorescence microscope, as reported elsewhere [128].

### 4.6. Measuring SOD, Gpx, and GSH Activity

The SOD activity was detected by the SOD activity assay kit (BioVision, Milpitas, CA, USA), the cell lysate was incubated with SOD enzyme solution, and the absorbance was read at 450nm. In addition, Gpx activity was detected by the Gpx activity colorimetric assay kit (BioVision), and the absorbance was read at 340 nm. In addition, the GSH level was detected using the Glutathione Colorimetric Assay Kit (BioVision), and the cell lysate was combined with 5,5′-dithio-bis(2-nitrobenzoic acid) (DTNB) at 412 nm. We read the yellow absorbance.

### 4.7. Statistical Analysis

All data are presented as the mean ± SEM of three independent experiments. We determined significance using an unpaired one-way ANOVA (ANOVA) followed by a SigmaPlot 12.5. The significance criterion was set at *p* < 0.001. If necessary, differences between groups were assessed with a Student’s *t*-test or one-way analysis of variance (One-way ANOVA).

## Data Availability

Not applicable.

## Supplementary Materials

The following supporting information can be downloaded at: https://www.mdpi.com/article/10.3390/ijms231911678/s1.
