# Supplementary material for: Resveratrol Mitigates Oxygen and Glucose Deprivation-Induced Inflammation, NLRP3 Inflammasome, and Oxidative Stress in 3D Neuronal Culture
Source: Int J Mol Sci. 2022 Oct 2;23(19):11678. doi: 10.3390/ijms231911678 (PMC9570351; doi:10.3390/ijms231911678)
Supplement: Supplementary file 1 [file ijms-23-11678-s001.zip › ijms-1905443-supplementary.pdf]

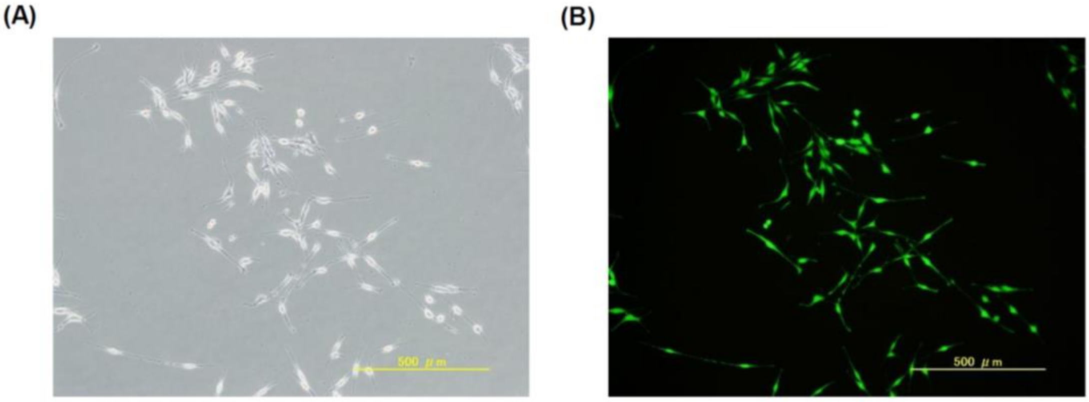

Figure S1. Cell morphology and expression of neuronal marker MAP2 in SH-SY5Y cells.
(A) Cell morphology of SH-SY5Y cells. (B) Immunostaining of the cells was performed using an anti- MAP2 antibody. MAP2 was visualized using Avidin- Alexa Fluor® 488 (green)-conjugated secondary antibody. A representative image of three independent experiments is shown. Scale bar: 500 μm.

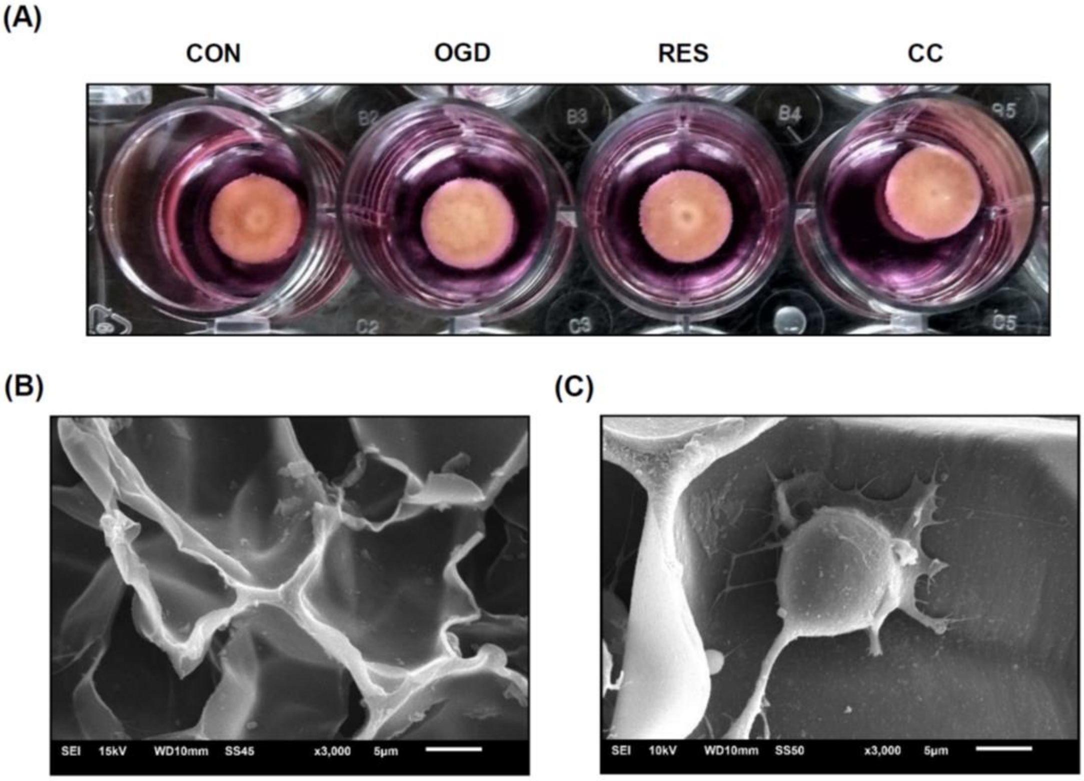

Figure S2. Images of the SH-SY5Y cells on a 3D gelatin scaffold.
SH-SY5Y cells were exposed with OGD on a 3D gelatin scaffold for 24 h, then treated with the indicated reagents (10 μM Resveratrol or 10 μM Compound C) for another 48 h. (A) Representative photographs of 3D condition show SH-SY5Y cells with gelatin scaffold (height: 2mm, diameter: 8mm). A scanning electron microscope (SEM; JEOL JSM6390LV) was used to determine the morphology of gelatin scaffold (B) or SH-SY5Y cells within the gelatin scaffold (C). A representative image of three independent experiments is shown. Scale bar: 5 μm.

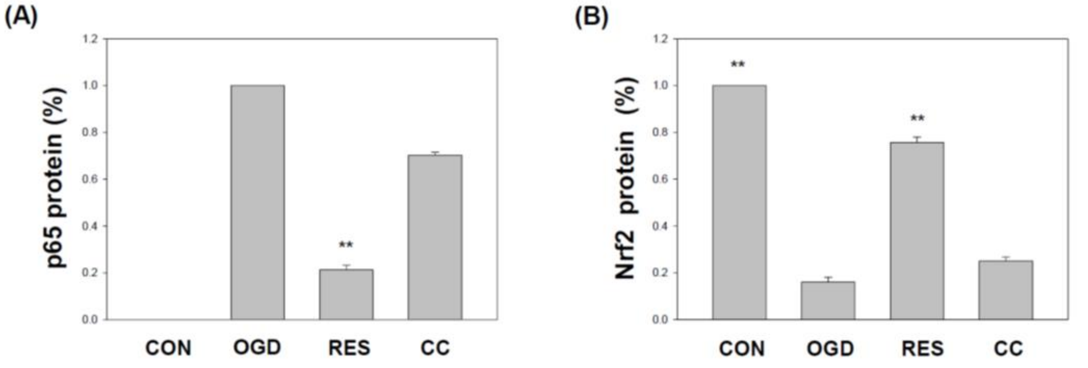

Figure S3. Quantification results of p65 and Nrf2 levels.

Collect nuclear components (20 μg per lane) from specified conditions and perform Western blot analysis. The level of p65 (A) and Nrf2 (B) protein is normalized with the corresponding internal control (lamin). Values are presented as the mean ± SEM values from three independent experiments. Specific comparison to the indicated SH-SY5Y cells with OGD **P<0.001 vs. cells with OGD.

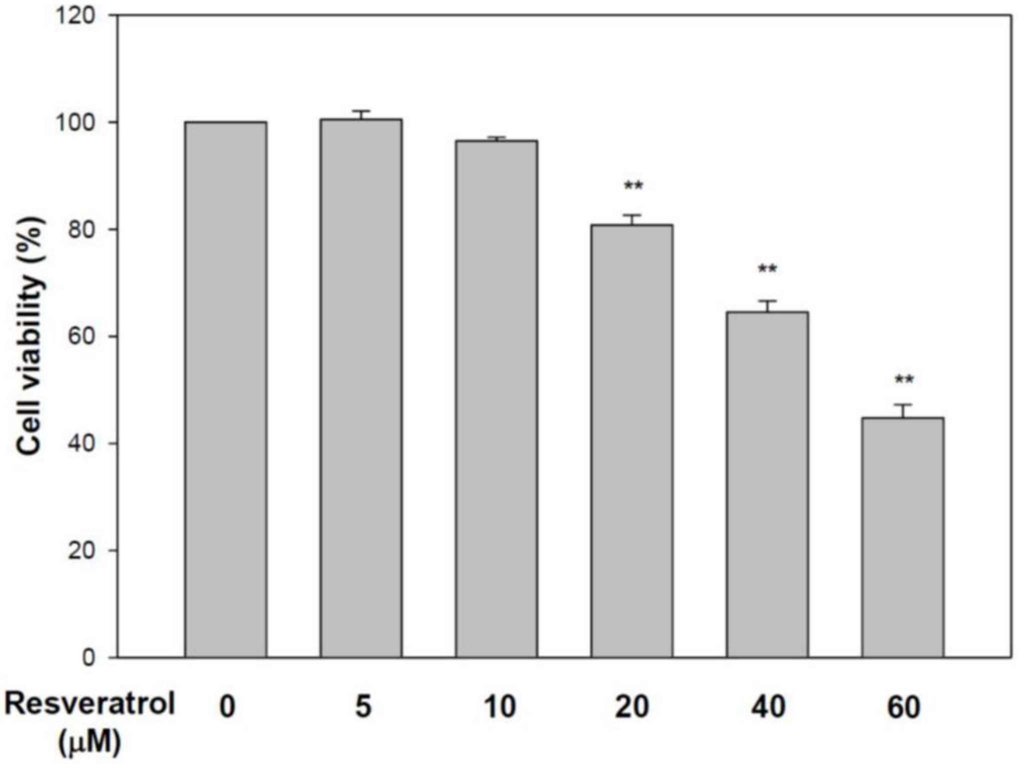

Figure S4. Effects of resveratrol on SH-SY5Y cells.

SH-SY5Y cells were treated with resveratrol (5, 10, 20, 40, and 60 μM) on a 3D gelatin scaffold for 48 h, and cell viability was detected by MTT assay. Values are expressed as the mean ± SEM values from three independent experiments. ** Specific comparison to the indicated cells with no treatment, P<0.001 vs. control group. One-way ANOVA).
